# Morning vs. evening: the role of exercise timing in enhancing fat oxidation in young men

**DOI:** 10.3389/fphys.2025.1574757

**Published:** 2025-04-23

**Authors:** Hao Lan, Kaibin Wu, Chunyun Deng, Songtao Wang

**Affiliations:** School of Physical Education and Sports Science, South China Normal University, Guangzhou, China

**Keywords:** timing exercise, energy expenditure, aerobic exercise, fat oxidation, circadian

## Abstract

**Objective:**

This study aimed to investigate the acute effects of exercise timing (morning vs. evening) on fat oxidation and energy expenditure in young men, with a focus on interactions between exercise and meal timing.

**Methods:**

Eighteen male college students (23.47 ± 2.11 years) completed a randomized crossover trial under five conditions: sedentary control (SC), exercise before breakfast (EBB), exercise after breakfast (EAB), exercise before dinner (EBD), and exercise after dinner (EAD). Indirect calorimetry (COSMED K5) measured substrate utilization during exercise, post-exercise recovery (0–4 h), and the following morning. Total exercise volume (running distance) was standardized, and energy expenditure was normalized to body weight (kcal/kg).

**Results:**

During the sedentary control test, participants showed similar trends in total energy expenditure. Dring exercise, the EBB group demonstrated significantly higher fat expenditure compared to EAB (P < 0.05), EBD (P < 0.01), and EAD (P < 0.01). Morning exercise overall exhibited superior fat oxidation (P < 0.01). Post-exercise (0–4 h), EBB sustained elevated fat utilization (P < 0.01 vs. EBD/EAD), while EAD showed enhanced fat oxidation the following morning (P < 0.01 vs. EAB).

**Conclusion:**

The findings suggest that exercise timing may influence temporal patterns of fat oxidation, with morning fasting potentially favoring acute lipid utilization, while evening exercise appears to correlate with delayed metabolic adjustments. Although total energy expenditure remained comparable across conditions, the observed shifts in substrate allocation imply a possible circadian-sensitive modulation of energy partitioning. These preliminary observations underscore the need for further investigation to clarify the long-term physiological relevance of such timing-dependent metabolic responses.

## Introduction

The circadian rhythm serves as a fundamental regulator of energy expenditure, governing temporal variations in substrate utilization and energy expenditure (mchill et al., 2014; Aoyama and Shibata, 2020). At the molecular level, core clock genes orchestrate the transcriptional activity of metabolic enzymes through direct genetic and epigenetic mechanisms, thereby imposing circadian oscillations on pathways critical to carbohydrate and lipid oxidation (Nisa et al., 2018; Coomans et al., 2013). For instance, the rhythmic expression of Bmal1 and Clock genes regulates the activity of enzymes involved in fatty acid oxidation, creating diurnal patterns in energy substrate partitioning (Sato et al., 2022; Gabriel and Zierath, 2019). These molecular oscillations translate into systemic metabolic rhythms, with studies demonstrating that fat oxidation rates exhibit distinct temporal peaks during the active phase in humans (Iwayama et al., 2015; Schumacher et al., 2020).

Exercise, as a potent zeitgeber, interacts with circadian physiology to modulate metabolic outputs. Emerging evidence suggests that the timing of exercise may synchronize peripheral clocks and amplify circadian-regulated metabolic processes (Sato et al., 2022; Sato et al., 2019). However, current research predominantly focuses on exercise modality, intensity, and duration, with limited exploration of how exercise timing—relative to endogenous circadian phases and feeding-fasting cycles—shapes acute and sustained energy partitioning. This gap is particularly salient given the growing recognition of chrono-exercise as a strategy to optimize metabolic efficiency (Yu et al., 2015).

Recent advances in chronotherapy, such as time-specific interventions for blood pressure regulation, highlight the importance of temporal precision in modulating physiological outcomes (Cornelissen et al., 2025; Hermida et al., 2013). Translating this concept to exercise physiology, the interaction between exercise timing and meal timing may critically influence energy expenditure (Aoyama and Shibata, 2020). For example, morning exercise performed in a fasted state could enhance fat oxidation due to lower glycogen availability, whereas evening exercise might leverage circadian-enhanced enzymatic activity for sustained substrate utilization. Despite such hypotheses, the combined effects of exercise and meal timing on energy expenditure across multiple phases (exercise, recovery, and subsequent fasting) remain underexplored.

Building upon these insights, this study investigates how morning *versus* evening exercise, conducted before or after meals, modulates energy expenditure during and after moderate-intensity aerobic activity in healthy males. Using continuous indirect calorimetry (Cosmed K5), we examined: 1) immediate substrate partitioning during exercise; 2) post-exercise metabolic recovery (0–4 h); and 3) next-morning fasting metabolism. Our results demonstrate that morning exercise preferentially enhances fat oxidation during and after activity, while evening exercise primes metabolic flexibility in the post-absorptive state. These time-of-day effects are further modulated by meal timing, revealing a dynamic interplay between circadian biology and nutritional cues in regulating energy homeostasis. By delineating the temporal metabolic trade-offs associated with exercise timing, this work provides a mechanistic framework for optimizing chrono-exercise strategies to enhance metabolic efficiency in healthy populations.

## Materials and methods

### Subjects

**FIGURE 1:**
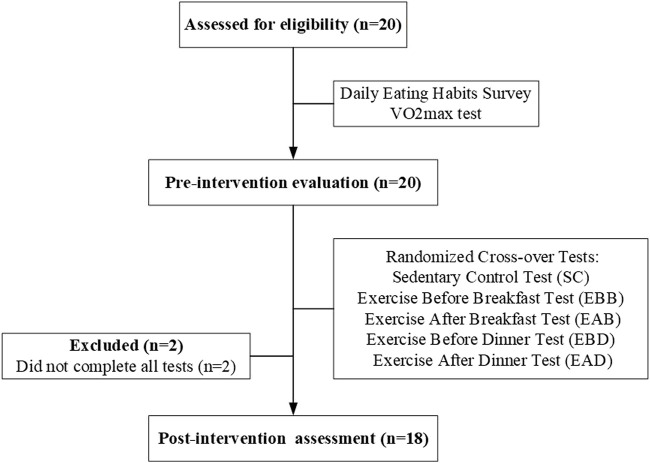
A simplified flowchart showing participant enrollment and grouping.

Between March and May 2020, 18 healthy male college students (mean age: 21.3 ± 1.5 years) were recruited through online platforms and campus flyers. All participants met the following inclusion criteria: 1) non-smokers with no alcohol consumption and stable daily routines (bedtime: 23:00–01:00; wake time: 07:00–08:00); 2) sedentary lifestyle defined by < 150 min/week of moderate-intensity physical activity or <75 min/week of vigorous activity (ACSM criteria); 3) absence of hypertension, cardiovascular diseases, or metabolic disorders; 4) no regular medication use; and 5) good sleep quality (Pittsburgh Sleep Quality Index, PSQI ≤5). Participants were excluded if they reported shift work, transmeridian travel in the prior month, or irregular sleep patterns (>2-h weekday-weekend sleep discrepancy). Written informed consent was obtained from all participants. Ethical approval was granted by the South China Normal University Institutional Review Board (No. SCNU-SPT-2020-002). Participant demographics, including anthropometric and baseline metabolic characteristics, are summarized in Table 1. Figure 1 illustrates the enrollment flowchart, detailing screening, exclusion, and final group allocation.

**TABLE 1:** Basic demographic characteristics information of participants.

Number	Age (years)	Height (cm)	Weight (kg)	BMI(kg/m2)	VO_2_max (mL/min/kg)
N = 18	23.47 ± 2.11	173.58 ± 4.71	66.81 ± 6.26	21.9 ± 1.80	48.19 ± 8.41

Abbreviations: BMI, body mass index; VO_2_max, Maximal Oxygen Consumption.

Data were presented as mean ± SD.

### Study design

This randomized crossover study evaluated the effects of exercise timing on energy expenditure using a standardized protocol. Participants’ maximal oxygen uptake (VO_2_max) was assessed using a metabolic gas analyzer (K5, COSMED, Italy) and the Bruce treadmill protocol (Howley et al., 1995; Guidetti et al., 2018; Chung et al., 2023). Participants exercised at incrementally increasing speeds (2.7–9.6 km/h) and inclines (10%–22%) until volitional exhaustion. The test was terminated if a VO_2_ plateau (<2 mL/kg/min fluctuation over three consecutive measurements), a respiratory quotient (RQ) > 1.1, or a rating of perceived exertion (RPE) ≥18 was reached (Ozemek et al., 2025). Treadmill speeds corresponding to 40% (for warm-up/cool-down) and 65% VO_2_max (for main exercise intensity) were calculated for individualized exercise prescriptions.

The experiment commenced 5–7 days post-VO_2_max testing, preceded by a 2-day dietary record to establish standardized control meals (breakfast: 8:10 a.m., lunch: 11:20 a.m., dinner: 5:00 p.m.) while maintaining habitual dietary patterns on non-trial days. Participants completed five randomized conditions: sedentary control (SC), exercise before breakfast (EBB: 6:50–7:40 a.m.), exercise after breakfast (EAB: 9:00–9:50 a.m.), exercise before dinner (EBD: 3:40–4:30 p.m.), and exercise after dinner (EAD: 6:00–6:50 p.m.), separated by ≥ 3-day washout periods prohibiting high-intensity activities.

Energy expenditure sampling occurred during exercise sessions (50 min total, comprising a 40-min main exercise, 5-min warm-up, and 5-min cool-down), post-exercise recovery (10-min sampling each hour for 4 h), and next-morning fasting (10-min measurement at 8:00 a.m.). Resting energy expenditure was measured during waking hours (7:00 a.m.–11:00 p.m.) after participants rested seated for at least 15 min. Sleep-phase data were not analyzed due to technical limitations. Energy expenditure was assessed via indirect calorimetry using a Cosmed K5 metabolic cart (COSMED, Italy) to continuously monitor oxygen consumption (VO_2_, L/min) and carbon dioxide production (VCO_2_, L/min). Carbohydrate and fat oxidation rates were calculated using validated stoichiometric equations derived from substrate-specific carbon-oxygen balance (Péronnet and Massicotte, 1991): Carbohydrate oxidation rate (g/min):4.585 × VCO_2_−3.226 × VO_2_, Fat oxidation rate (g/min):1.695 × VO_2_−1.701 × VCO_2_. Energy contributions were determined by multiplying oxidation rates by their respective energy equivalents (carbohydrate: 4 kcal/g; fat: 9 kcal/g) (LjungqvisT, 2011). Total energy expenditure (kcal/min) was calculated as the sum of carbohydrate and fat energy contributions. Substrate partitioning was further validated using the respiratory quotient (RQ = VCO_2_/VO_2_VCO_2_/VO_2_), where RQ ≈ 0.7 indicates predominant fat oxidation and RQ ≈ 1.0 reflects carbohydrate dominance (Jeukendrup and Wallis, 2005). Maximal oxygen uptake (VO_2_max), determined through incremental exercise testing to volitional exhaustion, was integrated to analyze intergroup differences in substrate utilization, with higher VO_2_max values correlating with enhanced fat oxidation efficiency. All values were normalized to individual body mass (kcal/kg) to account for inter-subject variability. Laboratory conditions were strictly controlled (25°C ± 2°C, 50%–60% humidity) to ensure consistency and accuracy. Scheduled hydration was provided, and accelerometry-verified sedentariness was maintained during non-exercise periods. Alcohol, caffeine, and high-fat diets were prohibited to minimize confounders, ensuring baseline sleep and activity patterns remained unchanged.

### Statistical analysis

Data analysis was performed using SPSS 27.0 software, with results expressed as mean ± standard deviation. One-way analysis of variance (ANOVA) was applied to determine differences in energy expenditure among exercise timing conditions (EBB, EAB, EBD, EAD) during exercise, post-exercise, and the following morning. Significant main effects (P < 0.05) were followed by Tukey HSD *post hoc* multiple comparisons. Normality was verified via Shapiro-Wilk test (P > 0.05), and homogeneity of variances was confirmed by Levene’s test (P > 0.05). A significance level of α = 0.05 was used for all statistical tests.

## Results

### Resting state

**FIGURE 2:**
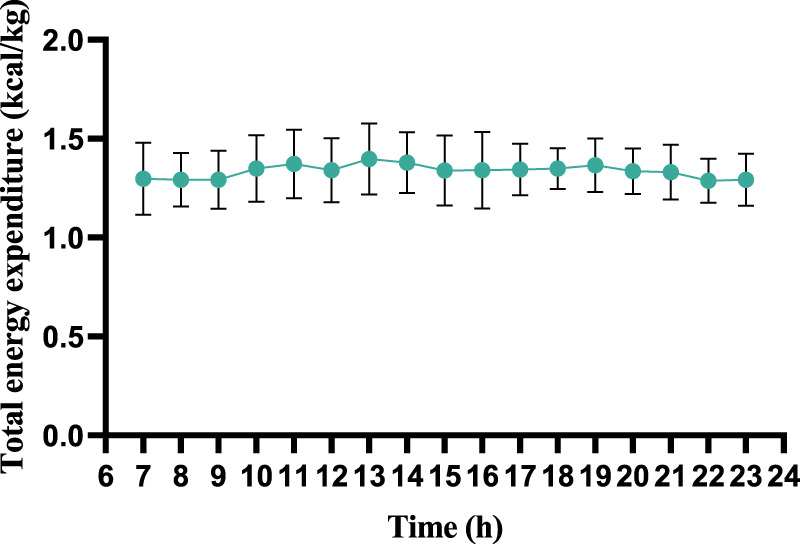
Wakeful-state total energy expenditure throughout the day (per 60 min).

During wakeful rest, participants exhibited consistent trends in energy expenditure and substrate metabolism, with no significant differences observed across all time points (refer to Figure 2). Regrettably, due to limitations of equipment and methodology, we were only able to observe energy expenditure during wakeful periods and were unable to further analyze energy changes during nighttime sleep.

This figure illustrates the dynamic changes in total energy expenditure across a 7–23 h observation period. The vertical axis represents total energy expenditure, and the horizontal axis indicates time (hours). Error bars reflect measurement variability, with data points demonstrating relatively stable energy expenditure throughout the timeframe, showing only minor fluctuations. The results suggest consistent metabolic activity during the monitored period, aligning with the controlled experimental conditions (temperature: 25°C ± 2°C; humidity: 50%–60%). Sleep-phase data were excluded due to technical limitations.

### During exercise

**FIGURE 3:**
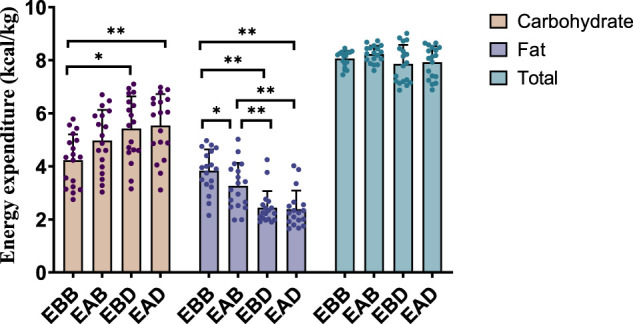
Energy expenditure during exercise (per 50 min).

**FIGURE 4:**
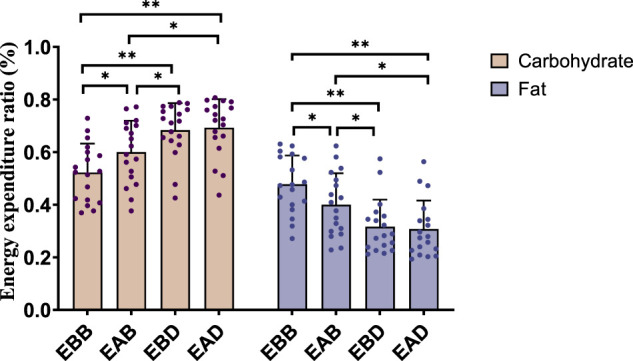
Energy expenditure ratio during exercise (per 50 min).

The results demonstrated significant differences in energy expenditure patterns among groups (refer to Figures 3, 4). From the perspective of energy expenditure, the carbohydrate expenditure in the EBB group was significantly lower than that in the EBD (P < 0.05) and EAD (P < 0.01) groups, whereas its fat expenditure was notably higher than in the EAB (P < 0.05), EBD (P < 0.01), and EAD (P < 0.01) groups. Morning exercise exhibited higher fat consumption compared to evening exercise (P < 0.01), while no significant difference was observed between EBD and EAD. Regarding energy expenditure ratios, the EBB group maintained a significantly higher fat ratio than EAB (P < 0.05), EBD, and EAD (both P < 0.01), with EAB also surpassing EBD (P < 0.05) and EAD (P < 0.05) in fat proportion. Conversely, carbohydrate ratios displayed an inverse trend. These findings suggest that exercise timing and group-specific interventions interactively modulate metabolic patterns, with morning sessions favoring fat-dominated energy utilization and the EBB group exhibiting pronounced advantages in lipid metabolism.

This figure combines bar graphs and scatter plots: 1) Bar graphs depict carbohydrate (brown), fat (purple), and total energy expenditure (blue) across EBB, EAB, EBD, and EAD groups (mean ± SD, unit: kcal/kg); 2) Scatter plots overlay individual data points to illustrate within-group variability; 3) Asterisks (*P < 0.05, **P < 0.01) denote significant intergroup differences derived from one-way analysis of variance (ANOVA) with Tukey HSD *post hoc* test. 4) Data were collected using a Cosmed K5 metabolic cart during a 50-min protocol comprising 40 min of exercise at 65% VO_2_max and 5-min warm-up/cool-down periods at 40% VO_2_max. All values were normalized to individual body weight (kcal/kg) to account for inter-subject mass variations.

This figure combines bar charts and scatter plots: 1) Bar charts depict carbohydrate (brown) and fat (purple) allocation ratios (mean ± SD, unit: %) across EBB, EAB, EBD, and EAD groups; 2) Scatter plots overlay individual data points on the bar charts to illustrate within-group variability; 3) Asterisks (*P < 0.05, **P < 0.01) denote significant intergroup differences derived from one-way analysis of variance (ANOVA) with Tukey HSD *post hoc* test.

### Post-exercise (0–4 h)

**FIGURE 5:**
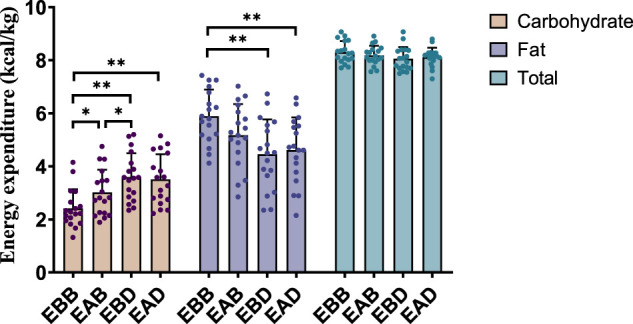
Energy expenditure during the recovery period after exercise (per 4 h).

**FIGURE 6:**
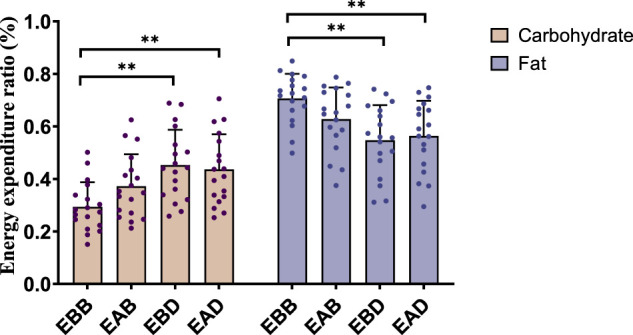
Energy expenditure ratio during the recovery period after exercise (per 4 h).

Post-exercise analysis (0–4 h) revealed distinct metabolic patterns among groups (refer to Figures 5, 6). From an energy expenditure perspective, the EBB group exhibited significantly lower carbohydrate expenditure compared to EAB (P < 0.05), EBD (P < 0.01), and EAD (P < 0.01), with EAB also showing reduced carbohydrate consumption relative to EBD (P < 0.05). Conversely, fat expenditure in the EBB group was markedly higher than in EBD (P < 0.01) and EAD (P < 0.01). In terms of energy expenditure ratios, the EBB group maintained a significantly higher fat proportion than EBD (P < 0.01) and EAD (P < 0.01), while no statistical differences in fat ratios were observed among EAB, EBD, and EAD groups. Carbohydrate ratios inversely mirrored this trend, with the EBB group displaying the lowest values. These findings indicate that the EBB group sustained a fat-dominated metabolic profile post-exercise, with divergent group-specific effects observed between absolute energy expenditure and proportional substrate allocation, suggesting time-dependent and group-regulated mechanisms in post-exercise energy substrate utilization.

This figure combines bar graphs and scatter plots: 1) Bar graphs depict carbohydrate (brown), fat (purple), and total energy expenditure (blue) across EBB, EAB, EBD, and EAD groups (mean ± SD, unit: kcal/kg); 2) Scatter plots overlay individual data points to illustrate within-group variability; 3) Asterisks (*P < 0.05, **P < 0.01) denote significant intergroup differences derived from one-way analysis of variance (ANOVA) with Tukey HSD *post hoc* test. 4) Data were collected using a Cosmed K5 metabolic cart during the post-exercise recovery phase, with breath-by-breath gas exchange measurements recorded hourly (10-min sampling intervals) over a 4-h period.

This figure combines bar charts and scatter plots: 1) Bar charts depict carbohydrate (brown) and fat (purple) allocation ratios (mean ± SD, unit: %) across EBB, EAB, EBD, and EAD groups; 2) Scatter plots overlay individual data points on the bar charts to illustrate within-group variability; 3) Asterisks (*P < 0.05, **P < 0.01) denote significant intergroup differences derived from one-way analysis of variance (ANOVA) with Tukey HSD *post hoc* test.

### Following morning

**FIGURE 7:**
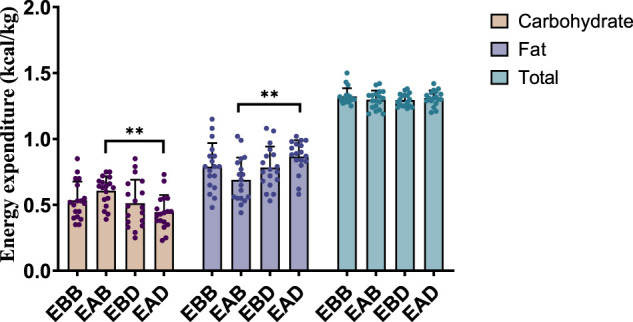
Energy expenditure in the following morning (per 60 min).

**FIGURE 8:**
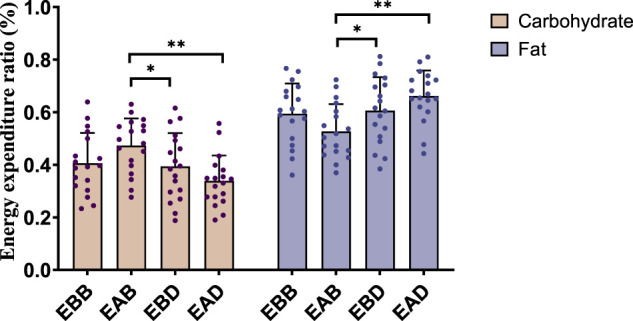
Energy expenditure ratio in the following morning (per 60 min).

The analysis of energy expenditure on the morning following exercise (refer to Figures 7, 8) revealed group-specific patterns. From an energy expenditure perspective, the EAB group exhibited significantly higher carbohydrate expenditure compared to EAD (P < 0.01) and lower fat expenditure than EAD (P < 0.01), with no significant differences in total energy expenditure across groups. Regarding energy expenditure ratios, the carbohydrate proportion in EAB was significantly higher than in EBD (P < 0.05) and EAD (P < 0.01), while its fat proportion was markedly lower than in EBD (P < 0.05) and EAD (P < 0.01), with no intergroup differences in total energy ratios. These findings suggest that energy substrate allocation during the post-exercise recovery phase is group-dependent, with EAB favoring carbohydrate utilization and EAD/EBD prioritizing fat metabolism, potentially influenced by temporal factors or metabolic memory mechanisms.

This figure combines bar graphs and scatter plots: 1) Bar graphs depict carbohydrate (brown), fat (purple), and total energy expenditure (blue) across EBB, EAB, EBD, and EAD groups (mean ± SD, unit: kcal/kg); 2) Scatter plots overlay individual data points to illustrate within-group variability; 3) Asterisks (*P < 0.05, **P < 0.01) denote significant intergroup differences derived from one-way analysis of variance (ANOVA) with Tukey HSD *post hoc* test. (4) Data were collected using a Cosmed K5 metabolic cart during the following morning’s fasting period (10-min measurement at 8:00 a.m.).

This figure combines bar charts and scatter plots: 1) Bar charts depict carbohydrate (brown) and fat (purple) allocation ratios (mean ± SD, unit: %) across EBB, EAB, EBD, and EAD groups; 2) Scatter plots overlay individual data points on the bar charts to illustrate within-group variability; 3) Asterisks (*P < 0.05, **P < 0.01) denote significant intergroup differences derived from one-way analysis of variance (ANOVA) with Tukey HSD post hoc test.

## Discussion

Most life forms, including humans, have evolved with internal timing mechanisms (molecular clocks) that regulate physiological functions such as metabolism, sleep, and hormone levels in a near 24-h cycle (Kovac et al., 2009; Boivin and Cermakian, 2009). These circadian rhythms, while endogenous, are influenced by external factors like sleep patterns, daylight exposure, and meal timings (Covassin et al., 1979; Ketchum and Morton, 2007). The human body’s circadian rhythms play a crucial role in regulating metabolism, sleep, and hormone levels. Recent studies have shown that exercise timing can significantly impact energy expenditure and fat oxidation. Our study found that morning exercise, particularly before breakfast, was more effective in burning fat compared to evening exercise. The temporary energy shortage induced by morning exercise effectively boosts the body’s fat oxidation for the subsequent 24 h.

Diurnal variations in exercise responses are influenced by basal metabolic and physiological state differences between morning and evening sessions (Sato et al., 2019). Evening exercise demonstrates advantages in physical performance, with studies reporting superior exercise capacity (Drust et al., 2005) and reduced perceived exertion at comparable intensities (EzagourI et al., 2019). Afternoon/evening exercise may prioritize carbohydrate utilization over fat oxidation, potentially enhancing mechanical efficiency through optimized actin-myosin interactions (Ayala et al., 2021). onversely, morning fasting exercise specifically augments lipid metabolism. Pre-breakfast exercise amplifies fat oxidation efficacy compared to fed-state conditions (Iwayama et al., 2015), with demonstrated 24-h persistence in elevated fat-derived energy utilization (Farah and Gill, 2013; Bennard and Doucet, 2006). This temporal effect correlates with reduced postprandial triglyceride levels and enhanced systemic lipid mobilization, particularly in overweight populations (Edinburgh et al., 2020). While pre-meal exercise associates with greater energy expenditure than post-meal sessions (Edinburgh et al., 2020), these effects are substrate-specific rather than reflective of total energy balance. Our data demonstrate that exercise timing modulates substrate utilization without altering total energy expenditure. Morning exercise preferentially enhanced fat oxidation compared to evening sessions, whereas delayed metabolic adaptations following evening exercise suggest temporal compartmentalization of exercise effects. This dissociation between acute substrate partitioning and delayed metabolic responses highlights the need to consider circadian-specific mechanisms when interpreting exercise-induced metabolic adaptations.

Several limitations should be acknowledged in this study. First, the small sample size and exclusive focus on young male participants limit the generalizability of our findings to females, older adults, or individuals with metabolic diseases. Future studies should include more diverse populations. Second, the short 24-h measurement period prevents conclusions about long-term effects of exercise timing on body composition or metabolic health. Longer-term interventions are needed. Third, while we controlled exercise and meals, individual differences in sleep patterns, body clocks, and precise nutrient intake were not fully measured, which might influence the results. Fourth, we did not test high-intensity or anaerobic exercise, so the timing effects on fat burning in these exercise types remain unknown. Finally, the lack of blood tests for hormones or fat-related molecules limits our understanding of why morning exercise burned more fat. Future research should track these biological markers and combine different exercise types to confirm our findings.

## Conclusion

This study reveals that acute moderate-intensity aerobic exercise induces time- and group-dependent modulation of energy expenditure: morning fasting exercise (EBB group) significantly enhanced fat oxidation during exercise and the subsequent 4-h recovery, while evening exercise (EAD group) was associated with elevated fat metabolism the following morning, suggesting time-of-day-specific substrate allocation and potential metabolic memory effects. Despite comparable total energy expenditure across groups, metabolic flexibility was evident through dynamic shifts in substrate ratios (inverse trends between fat and carbohydrates), indicating energy homeostasis may be maintained via substrate reallocation independent of total energy balance. Future research should integrate molecular mechanisms (e.g., circadian pathways, hormonal fluctuations) and long-term follow-up to elucidate the formation and maintenance of metabolic memory, while quantifying the regulatory roles of diet, sleep, and other confounders on temporal metabolic adaptations.

## Data Availability

The raw data supporting the conclusions of this article will be made available by the authors, without undue reservation.
